# Postoperative CA19-9 Change Is a Useful Predictor of Intrahepatic Cholangiocarcinoma Survival following Liver Resection

**DOI:** 10.1155/2015/298985

**Published:** 2015-12-29

**Authors:** Tae Yoo, Sang-Jae Park, Sung-Sik Han, Seong Hoon Kim, Seung Duk Lee, Young-Kyu Kim, Tae Hyun Kim, Sang Myung Woo, Woo Jin Lee, Eun Kyung Hong

**Affiliations:** ^1^Department of Surgery, Hallym University College of Medicine, 7 Keunjaebong-gil, Hwaseong-si, Gyeonggi-do 445-907, Republic of Korea; ^2^Center for Liver Cancer, National Cancer Center, 111 Jungbalsan-ro, Ilsandong-gu, Goyang-si, Gyeonggi-do 410-769, Republic of Korea

## Abstract

*Background*. To investigate the clinical significance of the perioperative CA19-9 change for predicting survival in intrahepatic cholangiocarcinoma (ICC) patients treated with surgical resection.* Methods*. We retrospectively reviewed the data from 74 ICC patients treated with surgical resection between April 2001 and July 2010. Perioperative CA19-9 (preoperative level, postoperative lowest level, and level at recurrence) levels were analyzed for patient distribution and survival.* Results*. Before surgery, there were 45 patients who had high preoperative CA19-9 levels (>37 U/mL) and 29 who had normal levels (≤37 U/mL). Of 45 patients with high CA19-9 levels, 34 had normalized CA19-9 levels after resection and 11 had persistently high levels. Of 34 patients with normalized CA19-9 levels, 18 showed recurrence. Of 29 patients with normal preoperative levels, 15 showed recurrence. Multivariate analysis presented that old age (hazard ratio [HR] = 3.881, *p* < 0.01), persistently high postoperative CA19-9 level (HR = 4.41, *p* < 0.001), perineural invasion (HR = 3.073, *p* = 0.01), narrow resection margin (HR = 3.152, *p* = 0.05), and lymph node metastasis (HR = 3.427, *p* = 0.02) were significant independent risk factors for survival.* Conclusions*. Patients who have normalized CA19-9 levels postoperatively have longer survival outcomes. Therefore, normalized postoperative CA19-9 may be a useful clinical marker for ICC survival.

## 1. Introduction

Intrahepatic cholangiocarcinoma (ICC) is the second most common primary hepatic cancer besides hepatocellular carcinoma but remains an uncommon and enigmatic disease [1]. ICC is one of the most biologically virulent malignant tumors and has a poor prognosis because it is frequently associated with lymph node involvement, intrahepatic metastasis, peritoneal dissemination, and/or infiltration into the bile duct and portal vein in the hepatic hilus [2, 3]. Although surgical resection offers the only chance of cure in ICC patients, resectability is still low and the 5-year survival rate is only 20–40% even in patients undergoing potentially curative resection [4]. Thus, many studies have been undertaken to investigate potential prognostic factors and their contribution to survival in patients who benefit from surgical resection.

Carbohydrate antigen 19-9 (CA19-9) is one of the most frequently studied prognostic factors which have been evaluated for diagnosis, survival, and recurrence in ICC patients. Previous studies have reported that CA19-9 expression is also prevalent in ICC^1^ and high preoperative CA19-9 level is independent dismal prognostic factor [1]. However, little is known about the serial changes in serum CA19-9 level even after surgical resection to recurrence and the relationships between perioperative serial serum CA19-9 levels and prognoses. Therefore, this study was conducted to investigate the significance of perioperative CA19-9 change from surgical resection in order to predict survival and recurrence in ICC patients.

## 2. Material and Methods

Between April 2001 and July 2010, a total of 99 patients who underwent surgical resection of ICC with curative intent and had a confirmed pathological diagnosis were recruited from our database. Then, 74 patients with complete data of perioperative CA19-9 levels, including preoperative CA19-9 (preopCA19-9), postoperative lowest CA19-9 (postopCA19-9), and CA19-9 levels at recurrence (recurCA19-9), were included in the study. Of the 74 patients, 54 (72.9%) were men and 20 (27.1%) were women, and their median age was 65 years (range, 27–82 years). Seventeen patients had viral hepatitis: 15 patients were positive for hepatitis B surface antigen and 2 for hepatitis C virus antibody. Hepatolithiasis was observed in 4 patients and liver cirrhosis in 15. PreopCA19-9 levels were increased above the normal value (reference value ≤37 U/mL) in 45 patients (60.8%) and were within the normal range in 29 patients (39.2%).

Preoperative diagnosis was based on a combination of radiologic staging, tumor makers testing (CA19-9 and CEA), and routine medical assessment. We restricted patients without elevated bilirubin (serum total bilirubin concentration <2.0 mg/dL) and/or no clinically evident cholangitis before and after surgery because insufficient control of the biliary obstruction could raise serum CA19-9 concentrations [5]. Routine radiologic examinations included dynamic computed tomography, dynamic magnetic resonance imaging, and positron emission tomography-computed tomography which had also been performed since 2006, when this modality became part of the coverage of the national health insurance of Korea. Preoperative biopsy was not routinely performed; however, all cases were confirmed by histology obtained from the resected specimens. Tumors were staged according to the UICC 6th edition TNM classification system. The residual tumor classification was used to define the presence or absence of residual tumor after resection: R0 signifies no residual tumor; R1 signifies microscopic positive margins; and R2 signifies macroscopic residual disease.

All 74 patients underwent liver resection with curative intent to obtain R0 resection. There were 6 trisectionectomies (right, 4; left, 2), 53 hemihepatectomies (right, 33; left, 20), 5 sectionectomies, and 10 segmentectomies. Combined resection of the extrahepatic bile duct was performed on 5 patients in whom the tumors invaded the extrahepatic bile duct. Lymph node dissection was performed on 51 patients (68.9%), and the policy of lymph node dissection in ICC surgery at our institute was previously described elsewhere [6]. Patients were closely followed up every 3-4 months and underwent tumor marker testing, chest radiography, and computed tomography every 6 months. Adjuvant therapy was typically not used in patients who underwent complete resection. However, adjuvant chemotherapy (5FU or Gemcitabine regimen) with or without radiotherapy was performed in selected high risk patients who had lymph node positive, multiple tumors. Sites of initial recurrence after resection were determined by review of follow-up imaging data.

### 2.1. Perioperative CA19-9 Levels

**Figure 1:**
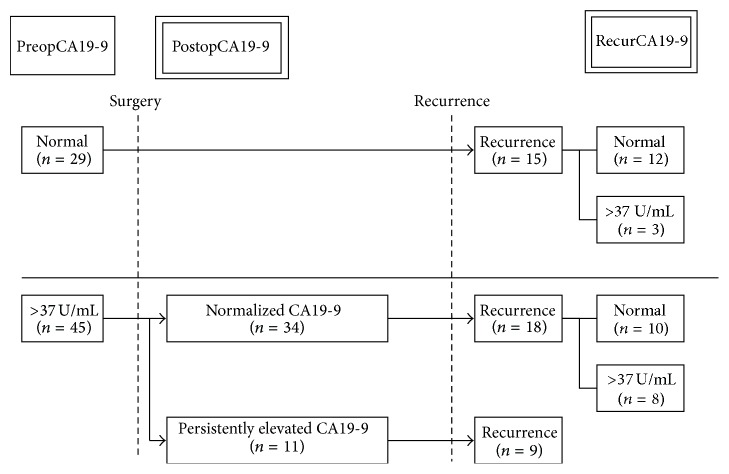
Patient distribution according to perioperative CA19-9 levels (*n* = 74).

Figure 1 shows patient distribution according to perioperative CA19-9 levels. Patients were stratified into 2 groups based on preopCA19-9: those with normal CA19-9 (≤37 U/mL, *n* = 29) and those with elevated CA19-9 (>37 U/mL, *n* = 45) before resection. After resection, we chose the postopCA19-9 level as the lowest level during follow-up period in order to allow adequate time for recovery from surgery or any adjuvant therapy. Forty-five patients with elevated preopCA19-9 were stratified into 2 groups based on the postopCA19-9 nadir.

We also checked CA19-9 at tumor recurrence during the follow-up period. When disease progression was confirmed by repeated imaging studies, the date of the first detection of a suspicious radiologic finding was recorded as the date of the initial disease recurrence and radiologic evidence of tumor recurrence was accepted as a criterion of recurrence even if a patient did not undergo a biopsy. Twenty-seven of the 45 patients with elevated preopCA19-9 recurred, and 15 of the 29 patients with normal preoperative levels recurred.

### 2.2. Statistical Analysis

The data on survival was obtained from the medical records of the patients. Survival analysis was undertaken using the Kaplan-Meier method, with group comparisons using log-rank analysis. To identify the influencing factors related to survival, the clinicopathological factors, age, sex, hepatitis B virus status, preopCA19-9 (37 U/mL), postopCA19-9 (37 U/mL), tumor size (5 cm), vascular invasion, perineural invasion, lymphatic invasion, intrahepatic metastasis, resection margin, lymph node metastasis, and adjuvant therapy, were analyzed by Cox's proportional hazard regression with forward stepwise technique. Demographics and clinicopathologic characteristics of the patients were compared between the groups using Student's *t*-test for continuous variables and using contingency table analysis (chi-square test or Fisher exact test when appropriate) for categorical variables. All statistical analyses were performed using STATA, version 10.1 for Windows. Statistical test results were two-sided, and a *p* value of ≤0.05 was considered statistically significant.

## 3. Results

The overall median survival time (MST) was 37.2 months (range, 6–95) and estimated rates of the overall survival at 1, 3, and 5 years were 72.2%, 51.5%, and 31.1%, respectively.

### 3.1. Perioperative CA19-9 Levels

#### 3.1.1. PreopCA19-9

**Figure 2:**
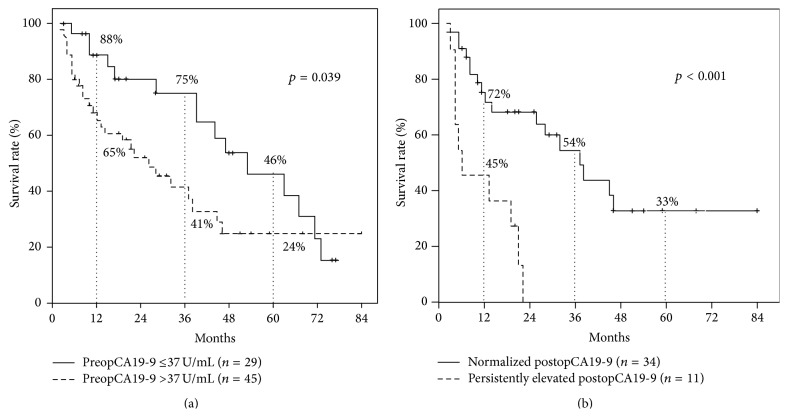
(a) Overall survival of intrahepatic cholangiocarcinoma patients according to preoperative CA19-9 levels (*n* = 74). (b) Overall survival according to postoperative CA19-9 levels in intrahepatic cholangiocarcinoma patients with elevated preoperative CA19-9 levels (*n* = 45).

Of the 74 patients, patients with normal preopCA19-9 had better survival than those with elevated CA19-9 levels (MST = 47 versus 22, *p* = 0.039) (Figure 2(a)).  Table 1 shows the comparison of clinicopathologic factors according to preopCA19-9 (preopCA19-9 >37 U/mL versus ≤37 U/mL). Patients with preopCA19-9 ≤37 U/mL had a higher positivity for HBsAg (*p* = 0.02) and had a tendency toward smaller tumors, less lymphatic invasion, less lymph node metastases, longer resection margins, and earlier T-stages, although statistically not significant (Table 1).

#### 3.1.2. PostopCA19-9

Of the 45 patients with elevated preopCA19-9 levels, patients with normalized postopCA19-9 had better survival than those with persistently elevated postopCA19-9 (MST = 43 versus 11 months, *p* < 0.001) (Figure 2(b)). Microvascular/perineural invasion was more frequent in patients with elevated postopCA19-9 (*p* = 0.034/*p* = 0.048) (Table 2).

#### 3.1.3. RecurCA19-9

**Figure 3:**
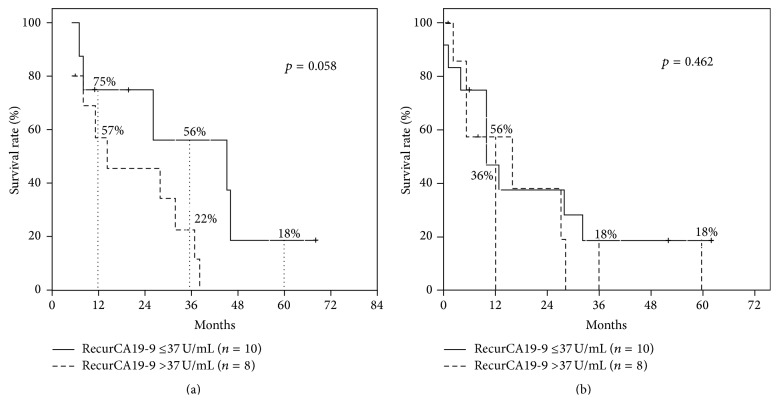
(a) Overall survival after resection according to recurrent CA19-9 levels in intrahepatic cholangiocarcinoma patients with normalized postoperative CA19-9 levels (*n* = 34). (b) Recurrence-free survival according to recurrence CA19-9 levels in intrahepatic cholangiocarcinoma patients with normalized postoperative CA19-9 levels (*n* = 34).

43 (58.1%) of the 74 patients recurred during follow-up period: 30 (69.8%) patients had intrahepatic recurrence, 4 (9.3%) had peritoneal dissemination, 3 (7%) had bone metastasis, 3 (7%) had lymph node metastasis, 2 (4.6%) had lung metastasis, and 1 (2.3%) had skin metastasis. Recurrence at resection site was observed in 11 of 30 patients with intrahepatic recurrence and rest of patients had hepatic recurrence at nonresection site. Of the 11 patients whose postopCA19-9 levels were still elevated, 9 recurred and died of the recurrence within 2 years (range, 6–22 months). Of the 34 patients with normalized postopCA19-9 levels, 8 with elevated recurCA19-9 levels showed marginal survival difference from 10 patients with normal recurCA19-9 levels after resection (MST = 14 versus 45, *p* = 0.058) (Figure 3(a)). However, in disease-free survival, there was no survival difference between two groups (MST = 4 versus 10, *p* = 0.126/MST = 10 versus 16, *p* = 0.462) (Figure 3(b)). Perineural invasion was more frequent in patients with elevated recurCA19-9 than in those with normalized postopCA19-9 levels (*p* = 0.042) (Table 3). Of the 29 patients with normal preopCA19-9 levels, 15 recurred. However, patients with normal preopCA19-9 levels showed no survival difference depending on recurCA19-9 levels (MST = 53 versus 39, *p* = 0.359).

### 3.2. Risk Factors in ICC Patients for Overall Survival

Univariate analysis showed that old age, high preopCA19-9 and postopCA19-9, major vessel invasion, perineural invasion, lymphatic invasion, narrow resection margin, and lymph node metastasis were risk factors in ICC patients for overall survival. Multivariate analysis showed that old age (hazard ratio [HR] = 3.881, 95% confidence intervals [95% CI] = 1.301–11.581, *p* < 0.01), persistently elevated postopCA19-9 levels (HR = 4.41, 95% CI = 2.01–6.72, *p* < 0.001), perineural invasion (HR = 3.073, 95% CI = 1.255–7.521, *p* = 0.01), narrow resection margin (HR = 3.152, 95% CI = 1.115–8.908, *p* = 0.05), and lymph node metastasis (HR = 3.427, 95% CI = 1.346–8.723, *p* = 0.02) were significant independent risk factors for ICC survival (Table 4).

## 4. Discussion

We limited our investigation to ICC patients who underwent radical resection with curative intent and had all data of preoperative, postoperative CA19-9 levels and CA19-9 levels at recurrence. In particular, after resection, we chose the postopCA19-9 level as the lowest level in order to allow adequate time for recovery from surgery or any adjuvant therapy prior to the time when an increase in CA19-9 levels was able to explain disease recurrence.

Many authors have described the associations between elevated preopCA19-9 levels and poor outcomes, in biliary malignancy, using different value cutoffs. Jan et al. have reported that high preopCA19-9 levels (>37 ng/dL) significantly influence the overall survival of ICC patients after hepatic resection [7]. It has been documented that preopCA19-9 levels are useful for the diagnosis of ICC patients associated with primary sclerosing cholangitis and that elevated CA19-9 levels are related to tumor burden. Ohtsuka et al. have also demonstrated that an increased preopCA19-9 level (>1000 units/L) is a negative prognostic factor [8]. In this study, patients with normal preopCA19-9 had significantly longer survival than those with elevated levels (MST = 47 versus 22, *p* = 0.039). Although there is no consensus regarding the predictability of preopCA19-9 for patient prognosis, our study has shown that even slightly elevated levels (>37 U/mL) appear to be associated with poorer outcomes. However, in our multivariate analysis, preopCA19-9 was not independent risk factor for survival. Furthermore, when we compared the clinicopathologic factors between patients with elevated and normal preopCA19-9 levels, patients with preopCA19-9 ≤37 U/mL had a higher positivity of HBsAg (*p* = 0.017).

We investigated changes in postopCA19-9 levels after surgery in patients with elevated preopCA19-9 levels. Although we assume that normalized postopCA19-9 would be favorable prognostic factor after curative resection of ICC, there is little information about changes in postopCA19-9 level for detecting ICC patients. On the contrary, some numerous studies on the relationship between pancreatic cancer and postopCA19-9 levels have been conducted to predict survival. Ferrone et al. reported that the strongest predictive levels of pre- and postopCA19-9 were 1,000 and 200 U/mL, respectively [9]. Decreased CA19-9 levels after surgery have been shown to be a strong predictor of the overall survival. Abdel-Misih et al. reported that the median overall survival rate was shorter in patients with persistent CA19-9 levels than in those with normalized CA19-9 levels (10.8 versus 23.8, *p* < 0.001) [10]. Uenishi et al. have indicated that persistently elevated CA19-9 is a single significant negative predictor of survival (hazard ratio = 2.20; *p* = 0.002) [11]. In this study, postopCA19-9 levels normalized after resection in 34 patients, while the levels remained persistently elevated in 11 patients. According to survival analysis, patients with failure of CA19-9 levels to normalize after resection had poorer survival rates than those with normalized CA19-9 levels (*p* < 0.001). Multivariate analysis also showed that persistently high postopCA19-9 was significant independent risk factors in ICC patient for overall survival. Therefore, postopCA19-9 may be more important factor in assessing the survival after resection, although preopCA19-9 concentration is important in the preoperative assessment of risk of survival. Moreover, high incidence of recurrence was observed in elevated postopCA19-9 compared with normalized postoperative levels. In group with persistently high postopCA19-9, 9 of 11 (81.1%) patients had recurrence after resection, while 18 of 34 (52.9%) patients with normalized postopCA19-9 had recurrence. Therefore, patients with high postopCA19-9 might be eligible for aggressive adjuvant chemotherapy and irradiation regardless of lymph node metastasis or resection margin status.

We also investigated the relationships between recurCA19-9 levels and survival rates. In 34 patients with elevated preopCA19-9 and normalized postopCA19-9 levels, only 8 of the 18 recurrent patients had elevated recurCA19-9 levels. Normalized postopCA19-9 patients with normal recurCA19-9 levels had marginally better survival than those with elevated recurCA19-9 levels (MST = 14 versus 45, *p* = 0.058). Whereas half of patients with normal preopCA19-9 levels had recurrence, most of them still had normal CA19-9 levels. This indicates that a considerable number of ICC patients with normal preopCA19-9 have normal recurCA19-9 levels. Therefore, patients with normal preopCA19-9 levels should be closely followed up with other tumor marker tests as well as radiologic studies.

The results of this study are subjected to some limitations. This is a retrospective cohort review of patients undergoing radical resection. By design, our study was based on 10 years of experience with treatment of ICC. With advances in surgical techniques and chemotherapy, there may be an element of lead time bias; patients treated more recently may have better survival rates because of lessons learned from more experience, better adjuvant therapy, and better supportive care. Despite these potential weaknesses, perioperative CA19-9 change was significant, and thus we believe that these perioperative CA19-9 levels may be predictors for prognosis in ICC patients treated with surgical resection.

## 5. Conclusions

Taken together, postoperative CA19-9 change is significant prognostic factors for predicting survival in ICC patients treated with surgical resection. Therefore, we suggest that this level should be measured in patients amenable to attempted curative resection. Moreover, postopCA19-9 level can be used to aid clinicians in the appropriate course of therapy. Because patients who do not have normalized CA19-9 levels postoperatively have shorter survival outcomes, accrual to clinical trials and continued or alternative therapy should be considered in such patients, even if radiographic evidence of disease is absent.

## Figures and Tables

**Table 1:** Comparison of clinicopathological factors according to preopCA19-9^†^ in ICC^‡^ patients (preopCA19-9 >37 U/mL versus preopCA19-9 ≤37 U/mL).

Factors	PreopCA19-9 ≤37 U/mL (*n* = 29)	PreopCA19-9 >37 U/mL (*n* = 45)	*p* value
Age (>65/≤65 years)	18/11	22/23	0.527
Sex (male/female)	19/10	25/20	0.359
Viral hepatitis B (yes/no)	10/19	5/40	0.017
Tumor size (>5/≤5 cm)	17/12	31/14	0.07
Major vascular invasion (yes/no)	3/26	8/37	0.169
Microvascular invasion (yes/no)	9/20	20/25	0.182
Perineural invasion (yes/no)	10/19	17/28	0.253
Lymphatic invasion (yes/no)	10/19	22/23	0.107
Intrahepatic metastasis (yes/no)	8/21	14/31	0.387
T stage, AJCC 6th (I, II/III, IV)	21/8	23/22	0.069
Lymph node metastasis (yes/no)	5/24	15/30	0.104
Resection margin (>1/≤1 cm)	24/5	29/16	0.093
Adjuvant therapy (yes/no)	10/19	13/32	0.399

PreopCA19-9^†^: preoperative CA19-9; ICC^‡^: intrahepatic cholangiocarcinoma.

**Table 2:** Comparison of clinicopathological factors according to postopCA19-9^§^ (normalized postopCA19-9 versus persistently elevated postopCA19-9) in patients with elevated preopCA19-9^†^ (*n* = 45).

Factors	Normalized postopCA19-9 (*n* = 34)	Persistently elevated postopCA19-9 (*n* = 11)	*p* value
Age (>65/≤65 years)	23/11	7/4	0.31
Sex (male/female)	23/11	6/5	0.353
Viral hepatitis B (yes/no)	3/31	2/9	0.355
Tumor size (>5/≤5 cm)	24/10	7/4	0.49
Major vascular invasion (yes/no)	5/29	2/9	0.443
Microvascular invasion (yes/no)	12/22	8/3	**0.034**
Perineural invasion (yes/no)	10/24	7/4	**0.048**
Lymphatic invasion (yes/no)	14/20	8/3	0.07
Intrahepatic metastasis (yes/no)	9/25	5/6	0.208
T stage, AJCC 6th (I, II/III, IV)	19/15	4/6	0.3
Lymph node metastasis (yes/no)	10/24	5/6	0.266
Adjuvant therapy (yes/no)	9/25	2/9	0.454
Resection margin (>1/≤1 cm)	23/11	7/4	0.565

PreopCA19-9^†^: preoperative CA19-9; postopCA19-9^§^: postoperative lowest CA19-9.

**Table 3:** Comparison of clinicopathological factors according to recurCA19-9^‖^ (recurCA19-9 >37 versus recurCA19-9 ≤37) in patients with elevated preopCA19-9^†^ and normalized postopCA19-9^§^ (*n* = 26).

Factors	RecurCA19-9 ≤37 U/mL (*n* = 10)	RecurCA19-9 >37 U/mL (*n* = 8)	*p* value
Age (>65/≤65 years)	8/2	3/5	0.145
Sex (male/female)	5/5	7/1	0.152
Viral hepatitis B (yes/no)	1/9	0/8	0.556
Tumor size (>5/≤5 cm)	7/3	7/1	0.558
Major vascular invasion (yes/no)	3/7	1/7	0.767
Microvascular invasion (yes/no)	4/6	6/2	0.051
Perineural invasion (yes/no)	4/6	7/1	**0.042**
Lymphatic invasion (yes/no)	4/6	4/4	0.520
Intrahepatic metastasis (yes/no)	5/5	6/2	0.367
T stage, AJCC 6th (I, II/III, IV)	4/4	13/6	0.352
Lymph node metastasis (yes/no)	5/5	6/2	0.367
Adjuvant therapy (yes/no)	5/5	4/4	1.00
Resection margin (>1/≤1 cm)	9/1	6/2	0.206
Intrahepatic recurrence (yes/no)	7/3	6/2	0.618
Extrahepatic recurrence (yes/no)	2/8	3/5	0.642
Disease-free survival (months)	10	4	0.126

PreopCA19-9^†^: preoperative CA19-9, postopCA19-9^§^: postoperative lowest CA19-9, and recurCA19-9^‖^: CA 19-9 levels at recurrence.

**Table 4:** Risk factors in ICC^‡^ patients for overall survival by Cox multivariate analysis.

Factors	Subgroups	*N*	HR (95% CI)	*p* value
Age (years)	>65		3.881 (1.301–11.581)	0.01
	≤65		Ref.	

PostopCA19-9^§^ (U/mL)	Elevated (>37)	11	4.41 (2.01–6.72)	<0.001
	Normalized (≤37)	63	Ref.	

Perineural invasion	(+)	45	3.073 (1.255–7.521)	0.01
	(−)	29	Ref.	

Resection margin	≤1 cm	43	3.152 (1.115–8.908)	0.05
	>1 cm	21	Ref.	

Lymph node metastasis	(+)	20	3.427 (1.346–8.723)	0.02
	(−)	54	Ref.	

ICC^‡^: intrahepatic cholangiocarcinoma; postopCA19-9^§^: postoperative lowest CA19-9.
